# Allyl Isothiocyanate Protects Acetaminophen-Induced Liver Injury via NRF2 Activation by Decreasing Spontaneous Degradation in Hepatocyte

**DOI:** 10.3390/nu12113585

**Published:** 2020-11-23

**Authors:** Min Woo Kim, Ju-Hee Kang, Hyun Jin Jung, Se Yong Park, Thu Han Le Phan, Hee Namgung, Seung-Yong Seo, Yeo Sung Yoon, Seung Hyun Oh

**Affiliations:** 1 Department of Anatomy and Cell Biology, College of Veterinary Medicine, Seoul National University, Seoul 08826, Korea; eastsea1203@gmail.com (M.W.K.); tpdyd2468@gmail.com (S.Y.P.); 2 College of Pharmacy, Gachon University, Incheon 21936, Korea; applekjh0503@hanmail.net (J.-H.K.); walkingjin86@gmail.com (H.J.J.); lephan.thuhan@gmail.com (T.H.L.P.); syseo@gachon.ac.kr (S.-Y.S.); 3 Department of Health Sciences and Technology, GAIHST, Gachon University, Incheon 21999, Korea; alice_lauren@naver.com

**Keywords:** allyl isothiocyanate (AITC), NRF2, acetaminophen (APAP, *N*-acetyl-*p*-aminophenol), hepatotoxicity

## Abstract

Acetaminophen (APAP) is one of the most frequently prescribed analgesic and anti-pyretic drugs. However, APAP-induced hepatotoxicity is a major cause of acute liver failure globally. While the therapeutic dose is safe, an overdose of APAP produces an excess of the toxic metabolite *N*-acetyl-*p*-benzoquinone imine (NAPQI), subsequently resulting in hepatotoxicity. Allyl isothiocyanate (AITC), a bioactive molecule in cruciferous plants, is reported to exert various biological effects, including anti-inflammatory, anti-cancer, and anti-microbial effects. Notably, AITC is known for activating nuclear factor erythroid 2-related factor 2 (NRF2), but there is limited evidence supporting the beneficial effects on hepatocytes and liver, where AITC is mainly metabolized. We applied a mouse model in the current study to investigate whether AITC protects the liver against APAP-induced injury, wherein we observed the protective effects of AITC. Furthermore, NRF2 nuclear translocation and the increase of target genes by AITC treatment were confirmed by in vitro experiments. APAP-induced cell damage was attenuated by AITC via an NRF2-dependent manner, and rapid NRF2 activation by AITC was attributed to the elevation of NRF2 stability by decreasing its spontaneous degradation. Moreover, liver tissues from our mouse experiment revealed that AITC increases the expression of heme oxygenase-1 (HO-1), an NRF2 target gene, confirming the potential of AITC as a hepatoprotective agent that induces NRF2 activation. Taken together, our results indicate the potential of AITC as a natural-product-derived NRF2 activator targeting the liver.

## 1. Introduction

Allyl isothiocyanate (AITC) is a naturally occurring compound found in Cruciferae family plants such as wasabi, and is reported to exert numerous biological effects, including anti-inflammatory, anti-neoplasia, anti-diabetic, and anti-microbial effects [1,2,3,4]. In Cruciferae plants, isothiocyanates are formed via the myrosinase-driven enzymatic hydrolysis of glucosinolates [5]. Recent papers have further reported that the microbiota metabolizes glucosinolates into isothiocyanates such as AITC [6,7], highlighting the importance of the dietary intake of Cruciferae plants and the function of isothiocyanates in the human body. Previously, we reported the beneficial effects of AITC on intestinal epithelium in the dextran sulfate sodium (DSS)-induced colitis model [8]. In the current study, we intend to further study the function of AITC in liver, since the absorbed AITC first enters the liver via portal circulation and is mainly metabolized here [9]. 

Acetaminophen (APAP, *N*-acetyl-*p*-aminophenol) is a widely used drug for painkilling and anti-pyretic effects in humans. Although the therapeutic dose of APAP is nontoxic, APAP overdose induces severe liver toxicity. APAP is mainly metabolized by glucuronidation and sulfation in the liver, while a minor portion is metabolized by cytochrome P450 2E1 (CYP2E1), producing the toxic metabolite *N*-acetyl-*p*-benzoquinone imine (NAPQI) [10]. NAPQI is detoxified in the liver by glutathione (GSH)-conjugation. However, excessive production of NAPQI depletes the GSH and leads to a toxic reaction with proteins, inducing oxidative stress and hepatocyte damage [11]. Thus, APAP overdose induces hepatic injury due to this excessive production of NAPQI. The APAP-induced hepatotoxicity murine model is widely applied to study functions of genes and antioxidant agents [12,13].

The nuclear factor erythroid 2-related factor 2 (NRF2), an essential transcription factor for antioxidant and detoxifying reactions, induces detoxifying genes when activated [14]. Under normal conditions, its natural repressor protein (Kelch-like ECH-associated protein 1 (Keap1)) binds to NRF2 and promotes ubiquitination of NRF2, resulting in proteosomal degradation in the cytoplasm [15]. When exposed to stimuli such as oxidative stress and activators, NRF2 is detached from Keap1 and escapes from ubiquitination and proteosomal degradation. Consequently, NRF2 moves into the nucleus and binds to the antioxidant response element (ARE), inducing the expression of various detoxifying and antioxidant genes, such as heme oxygenase-1 (HO-1) and NAD(P)H quinone oxidoreductase 1 (NQO1) [16]. According to knockout mouse studies, deficiency of NRF2 worsens the APAP-induced liver injury [17,18], thereby emphasizing the importance of NRF2 in the in vivo detoxifying process. Furthermore, NRF2 has been reported for its relevance in various liver diseases, including viral hepatitis, nonalcoholic steatohepatitis (NASH), and alcoholic liver disease [19,20,21,22]. Accordingly, NRF2 activation is highlighted as a potential therapeutic strategy to cure liver diseases [23].

Although there have been previous in vivo studies reporting the biological effects of AITC on liver, they mainly covered the effects of AITC on immune cell regulation and lipid accumulation [24,25]. In this work, we first investigate the beneficial effects of AITC using the APAP-induced hepatic injury model in order to verify the potential of AITC as a hepatoprotective agent activating NRF2. Furthermore, we confirm NRF2 activation by AITC in hepatocyte cell lines, and further explore the mechanism involved in NRF2 activation by AITC, demonstrating that AITC activates NRF2 by reducing spontaneous degradation of NRF2, and not by increasing transcription. Considering our results, we propose the potential of AITC as a natural NRF2 activator for liver protection.

## 2. Materials and Methods 

### 2.1. Cells and Materials

HepG2 and AML12 cell lines were obtained from the Korea Cell Line Bank (Seoul, Korea) and ATCC (Manassas, VA, USA), respectively. HepG2 was cultured in Roswell Park Memorial Institute (RPMI) 1640 medium supplemented with 10% fetal bovine serum (FBS) and penicillin/streptomycin (Welgene, Daegu, Korea). AML12 was cultured in DMEM: F12 medium supplemented with 10% FBS, penicillin/streptomycin, insulin, transferrin, selenium, and dexamethasone, according to the supplier’s recommendation. APAP was purchased from Sigma-Aldrich (St Louis, MO, USA), and primary antibodies for western blotting were obtained from several different vendors: Cleaved PARP (#9542) and pJNK (#9251, Cell Signaling Technology, Danvers, MA, USA), α-Tubulin (CP06) and GAPDH (CB1001, Merck Millipore, Darmstadt, Germany), lamin B (sc-6217) and Keap1 (sc-514914, Santa Cruz Biotechnology, Santa Cruz, CA, USA), NRF2 (ab62352, Abcam, Cambridge, MA, USA), and HO-1 (AF3776, R&D systems, Minneapolis, MN, USA).

### 2.2. APAP-Induced Liver Damage Model and Sample Analysis

Two sets of animal experiments were performed at Gachon University, in accordance with the procedures approved by the Institutional Animal Care and Usage Committee (IACUC) (GIACUC-R2019022, GIACUC-R2019044). Seven-week-old C57BL/6 mice were purchased from Koatech (Pyeongtaek, Korea). After acclimation for 7 days, mice were randomly divided into four groups (*n* = 5–10): (1) control group, (2) APAP-only treatment group, (3) low-dose AITC + APAP group, and (4) high-dose AITC + APAP group. After overnight fasting without depriving water, the mice were orally administered vehicle (corn oil) or AITC (25 or 50 mg/kg, low-dose and high-dose groups, respectively). Two hours later, the mice received either vehicle (PBS) or APAP 250 mg/kg intraperitoneally, followed by free access to food. Mice were sacrificed at 6 h, and blood and liver tissues were subsequently harvested for further studies. In another independent experiment, the mice were grouped and treated as described above, then sacrificed after 13 h of APAP exposure. Serum was isolated from blood samples, and analyzed with HITACHI 7180 to measure levels of aspartate aminotransferase (AST) and alanine aminotransferase (ALT). Formalin-fixed liver tissues were processed to produce paraffin blocks. The sectioned liver tissues (4 μm) were subjected to hematoxylin and eosin staining for microscopic observation [26].

### 2.3. RNA Isolation and RT-PCR

After treating the cells as described below, RNA was extracted using TRIzol (Invitrogen, Carlsbad, CA, USA) from cells or frozen liver tissues, according to the manufacturer’s instructions. The isolated RNA (2 μg) was used to synthesize complementary DNA (cDNA), by applying the cDNA synthesis kit from TaKaRa (Kusatsu, Japan). cDNA samples were subjected to PCR using primers specific to each target, as follows: mouse primers: HO-1, 5′-GCA CTA TGT AAA GCG TCT CC-3′ (forward) and 5′-TTG ACC TCA GGT GTC ATC TC-3′ (reverse); NQO1, 5′-CGG CTC CAT GTA CTC TCT TC-3′ (forward) and 5′-GGC TGC TTG GAG CAA AAT AG-3′ (reverse); tumor necrosis factor-α (TNF-α), 5′-ATA GCT CCC AGA AAA GCA AGC-3′ (forward) and 5′- CAC CCC GAA GTT CAG TAG ACA-3′ (reverse); GAPDH, 5′-AAG GGC ATC TTG GGC TAC ACT-3′ (forward) and 5′-TAC TCC TTG GAG GCC ATG TAG G-3′ (reverse); human primers: NRF2, 5′-CAA TGA TTC TGA CTC CGG CA-3′ (forward) and 5′-CAG GGG CAC TAT CTA GCT CT-3′ (reverse); GAPDH; 5′-GGT GAA GGT CGG TGT GAA CGG ATT T-3′ (forward) and 5′-AAT GCC AAA GTT GTC ATG GAT GAC C-3′ (reverse). Subsequent to the PCR reaction, the PCR products were analyzed with 2% agarose gel in tris-acetate/ethylenediaminetetraacetic acid (EDTA) buffer. The bands were quantified using ImageJ (NIH, Bethesda, MD, USA).

### 2.4. Western Blotting

After treating the cells as described above, total protein was extracted from cells with radioimmunoprecipitation assay (RIPA) buffer containing protease inhibitor and phosphatase inhibitor. Total protein from liver tissue was isolated after homogenization with the same buffer. For nuclear fractionation, we used the Nuclear Extraction Kit (Abcam, Cambridge, MA, USA), according to the manufacturer’s instructions. In the knockdown study, non-specific control siRNA or siRNAs targeting NRF2 (combination of 5′-AAG AGU AUG AGC UGG AAA AAC-3′ and 5′-GAG ACU ACC AUG GUU CCA A-3′) were applied to cells using RNAiMAX (Invitrogen, Carlsbad, CA, USA) according to the manufacturer’s instructions. In the stability evaluation study of NRF2, the HepG2 cells were pretreated with or without AITC (10 μM) for 1 h, and then exposed to cyclohexamide (10 μg/mL) for 15, 30, or 60 min. After quantification of total protein content with BCA assay kits (Pierce, Rockford IL, USA), equivalent amounts of protein (10 μg) were subjected to sodium dodecyl sulfate-polyacrylamide gel electrophoresis. The separated proteins were then transferred to polyvinylidene fluoride (PVDF) membrane, followed by blocking with 5% skim milk in TBS-T, and overnight incubation with primary antibodies. The next day, the probed membranes were incubated with horseradish peroxidase (HRP)-conjugated secondary antibodies, and detection was achieved using Absignal (Abclone, Seoul, Korea). The bands were quantified using ImageJ (NIH, Bethesda, MD, USA).

### 2.5. Immunocytochemistry

Immunocytochemistry was performed as previously described, with minimal changes [27]. Briefly, HepG2 cells seeded on coverslips were exposed to AITC for 1 h, and subsequently fixed and permeabilized with an acetone and methanol mixture (1:1) for 20 min at −20 °C. After blocking, the cells were incubated with primary antibody at 4 °C overnight, followed by incubation with Texas Red-labeled secondary antibody for 2 h at room temperature. Lastly, the coverslips were mounted with 4′,6-diamidino-2-phenylindole (DAPI) mounting medium for nuclear counterstaining, and images were obtained by confocal microscopy.

### 2.6. Statistical Analysis

All quantitative data are expressed as mean ± standard error of mean (SEM). Statistical analysis was performed with GraphPad Prism 6 (GraphPad Software, San Diego, CA, USA). One-way ANOVA test followed by Newman–Keuls test or unpaired *t* test (two-tailed) was used for analysis, and *p* values < 0.05 were considered statistically significant.

## 3. Results

### 3.1. AITC Pretreatment Attenuates APAP-Induced Hepatotoxicity In Vivo

**Figure 1:**
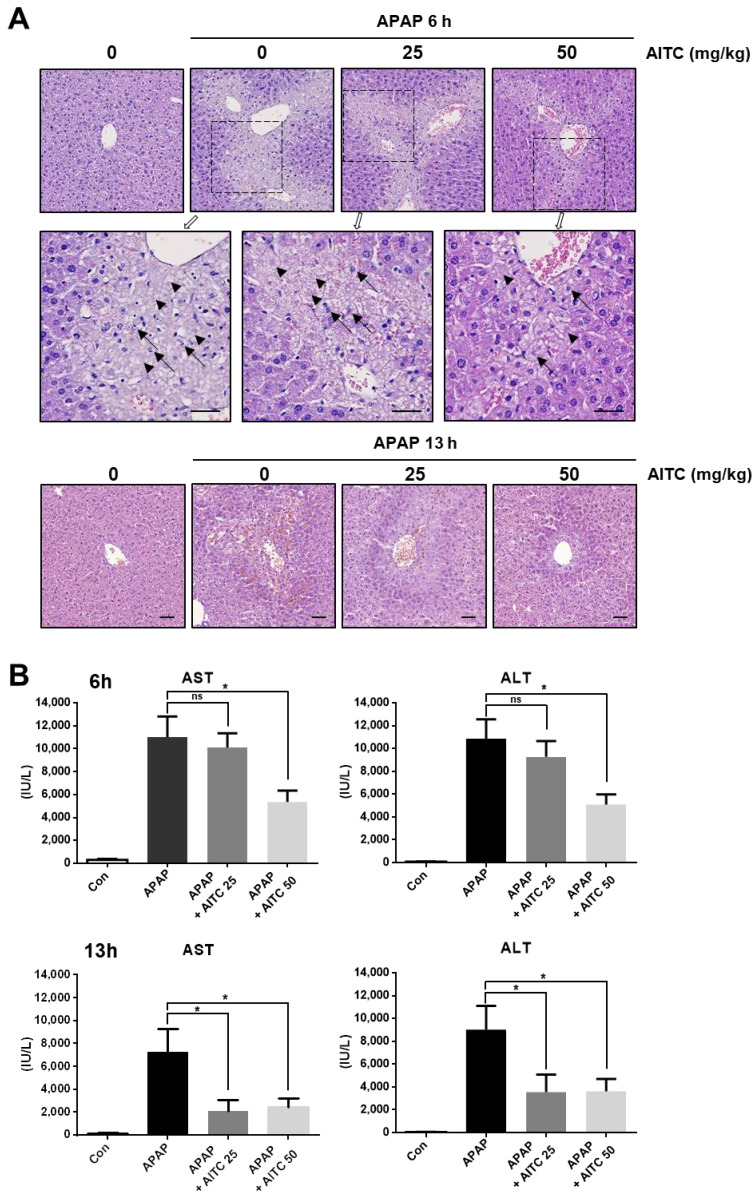
Attenuation of acetaminophen (APAP)-induced hepatotoxicity by allyl isothiocyanate (AITC) pretreatment. (**A**) Liver tissues from control mice and APAP-treated mice (upper: 6 h (boxed area is enlarged below), lower: 13 h) with or without pretreatment of AITC (25 or 50 mg/kg) were observed microscopically (H&E staining, scale bar 50 μm, arrow: karyorrhexis, arrow head: ghost cell). (**B**) Aspartate aminotransferase (AST) and alanine aminotransferase (ALT) levels were measured in serum samples (upper: 6 h, lower: 13 h). The data are presented as mean ± standard error of mean (SEM). One-way ANOVA was used for statistical analysis; ns, not significant; * *p* < 0.05.

**Figure 2:**
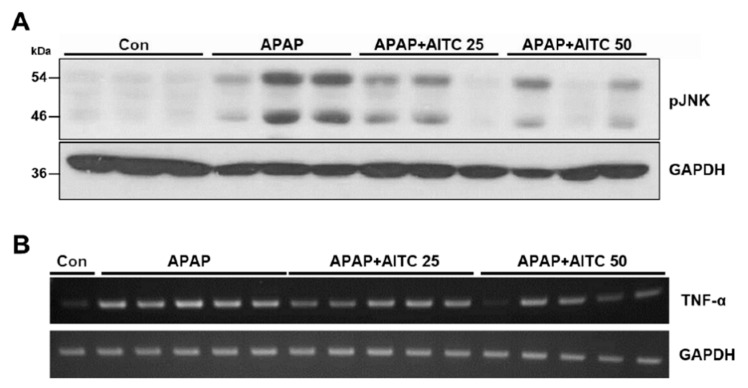
AITC pretreatment reduces APAP-induced phosphorylation of c-Jun N-terminal kinase (JNK) and tumor necrosis factor-α (TNF-α) expression. Immunoblotting was performed to evaluate the phosphorylation of JNK as an APAP-induced damage marker in liver tissues from (**A**) 13 h APAP treatment set. (**B**) TNF-α expression level was analyzed by RT-PCR in 13 h APAP treatment set.

To investigate whether AITC bestows protection to the liver from APAP-induced hepatotoxicity, mice were orally pretreated with AITC (25 or 50 mg/kg, in corn oil), and subsequently administered APAP (250 mg/kg, in PBS). We conducted two independent sets of animal experiments encompassing two time points after APAP injection (6 or 13 h). In both experimental sets, microscopic observation revealed that APAP treatment induces hepatocellular damage around the central vein (Figure 1A), which is characterized by pathologic features such as karyorrhexis and ghost cells. Conversely, liver sections from AITC high-dose group at 6 h post-APAP, and at both doses of AITC groups in the 13 h post-APAP, showed attenuation of hepatic injury as compared to tissues obtained from the APAP-only treatment groups (Figure 1A). Consistent with histologic observation, APAP injection significantly elevated serum levels of AST and ALT in both sets, which was significantly reversed by AITC pretreatment (except low-dose group at 6 h post-APAP) (Figure 1B), demonstrating the protective effect of AITC against APAP-induced liver damage. Additionally, immunoblotting analysis was conducted with liver tissues to detect the phosphorylation of c-Jun N-terminal kinases (JNKs), which is reported as the major molecule mediating hepatocyte death in the APAP overdose model (Figure 2A) [28]. We determined that there was increased phosphorylation of JNK in liver tissues when exposed to APAP for 13 h, whereas AITC pretreatment reduced the APAP overdose-induced JNK phosphorylation (Figure 2A), thereby indicating alleviation of pathogenesis by AITC. Although hepatocytes near the central vein are the primarily affected cells in the APAP overdose model, damage-associated molecular patterns (DAMPs) released from hepatocytes stimulate immune cells, inducing sterile inflammation at late time points [29]. Therefore, we assessed the TNF-α expression level by RT-PCR using liver tissues from the APAP 13 h set. Compared with the APAP-only treatment group, lower levels of TNF-α were determined in both the low- and high-dose AITC groups (Figure 2B). Taken together, results from the two animal experimental sets indicate that AITC attenuates the APAP-induced liver injury in vivo. 

### 3.2. AITC Induces NRF2 Activation and Its Target Gene Expression in Hepatocytes

**Figure 3:**
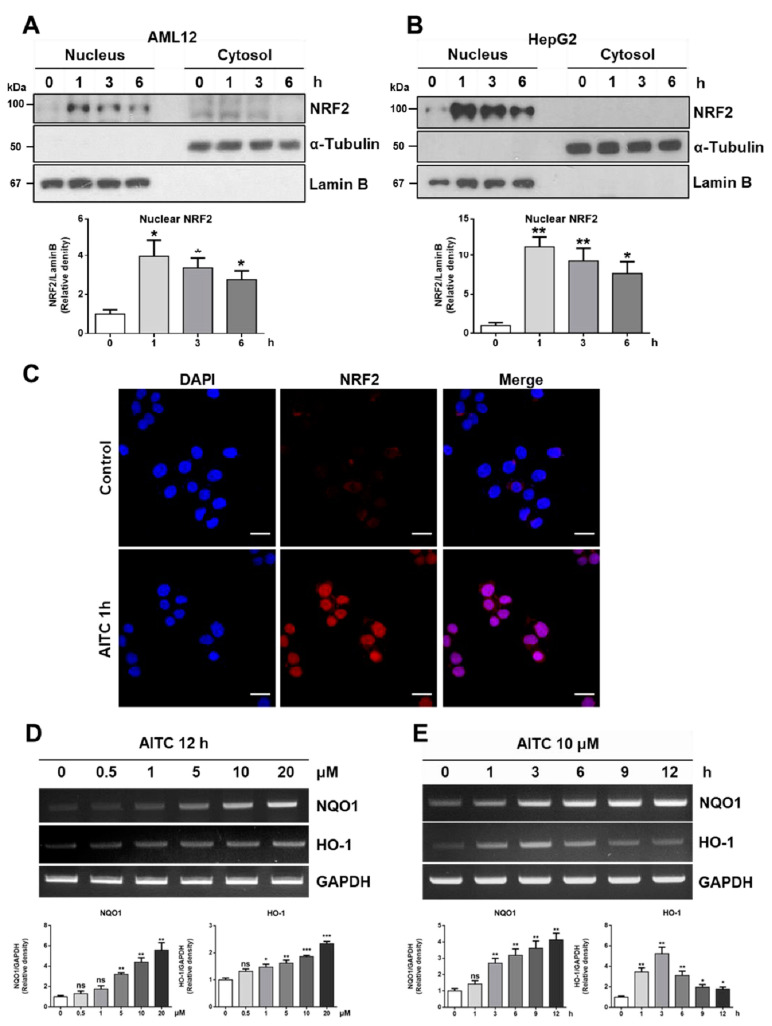
AITC induces nuclear translocation of nuclear factor erythroid 2-related factor 2 (NRF2) and expression of NRF2 target genes. After AITC treatment (10 μM) for indicated times (1~6 h), the nuclear fraction and cytosol fraction samples were examined by western blot analysis to evaluate the activation of NRF2 in (**A**) AML12 and (**B**) HepG2. (**C**) HepG2 cells were subjected to immunocytochemistry to visualize nuclear translocation after 1 h of treatment with AITC (scale bar 20 μm). (**D**,**E**) To evaluate NRF2 target gene expression, AML12 cells were exposed to several doses of AITC (0.5~20 μM) for 12 h, or 10 μM AITC for the indicated times. The mRNA obtained was analyzed by RT-PCR to detect NQO1 and HO-1 expressions. Three independent experiments showed consistent results, and values were obtained from three independent experiments. Unpaired *t* test was used for statistical analysis. ns, not significant; * *p* < 0.05, ** *p* < 0.01, *** *p* < 0.001 compared with control.

According to previous works, NRF2 activation is required to protect APAP-induced liver damage [17,18], and AITC is known to have an NRF2 activation effect [30]. Thus, we examined whether AITC can activate NRF2 in hepatocytes, using HepG2 and AML12 cell lines. After exposure to AITC (10 μM) for the indicated time (1~6 h), the nuclear fraction and cytosolic faction of cells were subjected to western blot in order to analyze the nuclear translocation of NRF2 (Figure 3A,B). We observed the AITC-induced nuclear translocation of NRF2 after only 1 h of exposure to AITC, which persisted until 6 h in both the HepG2 and AML12 cell lines (Figure 3A,B). The nuclear translocation of NRF2 after 1 h of treatment with AITC was further confirmed by subjecting the treated HepG2 cells to immunocytochemistry (Figure 3C). We next performed RT-PCR to check alterations in the expressions of NRF2 target genes using AML12 cells, which revealed both dose-dependent and time-dependent increases of NRF2 target genes such as NQO1 and HO-1, known for their importance in antioxidant reaction (Figure 3D,E). Collectively, these results indicate that AITC induces NRF2 activation and target gene expression in hepatocytes, thereby confirming the potential of AITC as an NRF2 activator targeting the liver. 

### 3.3. AITC Protects APAP-Induced Cell Damage via NRF2 Activation

**Figure 4:**
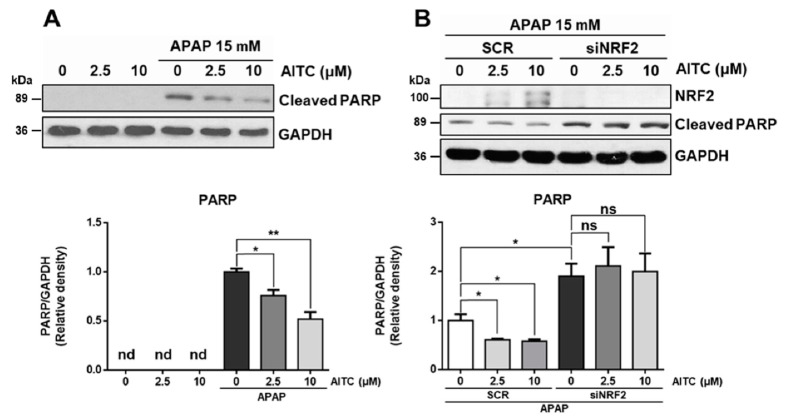
AITC ameliorates APAP-induced PARP cleavage in HepG2 through NRF2-dependent manner. (**A**) Cleaved PARP was analyzed by western blotting. HepG2 cells were incubated with or without AITC for 2 h, and subsequently exposed to 15 mM of APAP for 14 h. (**B**) To address NRF2 dependency, HepG2 cells transfected with scrambled siRNA (SCR) or siRNA against NRF2 (siNRF2) were exposed to the same schedule of treatment, followed by western blot analysis. Three independent experiments showed consistent results, and values were obtained from three independent experiments. Unpaired *t* test was used for statistical analysis. ns, not significant; * *p* < 0.05, ** *p* < 0.01.

Next, we examined the protective effect of AITC against APAP-induced cell damage, using the HepG2 cell line. Briefly, the HepG2 cell line was treated with APAP and AITC. Western blot of the treated cells revealed that exposure to 15 mM APAP for 14 h induced PARP cleavage (Figure 4A). Also, pretreatment of AITC (2.5 or 10 μM) for 2 h resulted in decreased PARP cleavage, thereby revealing the protective effect of AITC in hepatocytes. To determine the involvement of NRF2 in the protective effect of AITC, HepG2 cells were subjected to transient knockdown using siRNA (Figure 4B). Interestingly, NRF2 knockdown worsened the APAP-induced PARP cleavage, and the protective effect of AITC was diminished by NRF2 knockdown, indicating that the protective effect of AITC is mainly dependent on the NRF2 pathway in APAP-induced cell injury.

### 3.4. AITC Rapidly Activates NRF2 by Reducing Spontaneous Degradation

**Figure 5:**
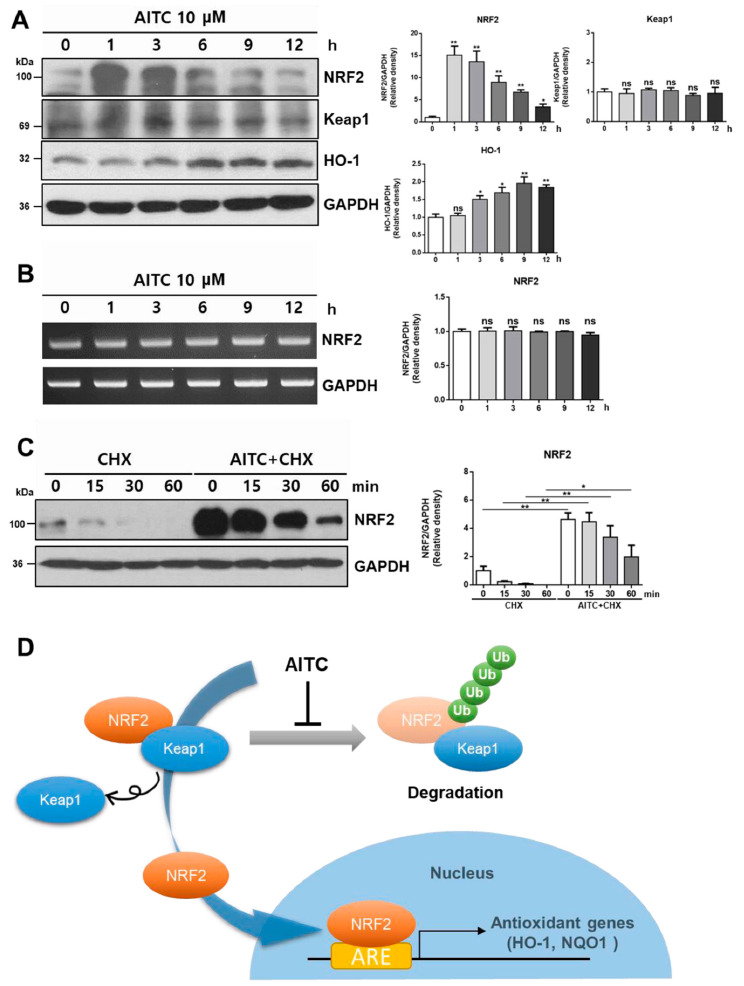
AITC quickly enhances NRF2 activity by reducing the spontaneous degradation of NRF2 and increasing stability, but not by regulating the transcription of NRF2. (**A**) Whole cell lysate protein samples, obtained after the incubation of HepG2 cells with AITC for 1~12 h, were subjected to western blotting. (**B**) RT-PCR analysis was conducted using samples from same conditions. (**C**) HepG2 cells were exposed to cyclohexamide (CHX, 10 μg/mL) for indicated times after 1 h of incubation with AITC in order to assess the effect of AITC on NRF2 degradations. (**D**) Scheme of NRF2 activation by AITC. Under normal conditions, NRF2 is degraded after polyubiquitination promoted by Keap1. In the presence of AITC, NRF2 escapes degradation and is translocated to the nucleus, inducing the expression of target genes by binding ARE. Three independent experiments showed consistent results, and values were obtained from three independent experiments. Unpaired *t* test was used for statistical analysis. ns, not significant; * *p* < 0.05, ** *p* < 0.01 compared with control.

We further investigated the mechanism of NRF2 activation by AITC. Treatment of HepG2 cells with 10 μM AITC quickly elevated the NRF2 level in whole cell lysates after just 1 h of exposure, simultaneous with a time-dependent increase of HO-1 and no remarkable change of Keap1 (Figure 5A). However, RT-PCR analysis revealed that AITC induced minimal alterations of NRF2 mRNA in HepG2 cells (Figure 5B), implying that rapid elevation of the NRF2 protein level in whole cell lysates is not attributed to transcriptional regulation. Therefore, we investigated the effect of AITC on spontaneous NRF2 degradation. To evaluate NRF2 degradation and its stability, we used the translational inhibitor, cyclohexamide. Exposure of HepG2 cells to cyclohexamide for the indicated time (15~60 min) resulted in the time-dependent decrease of NRF2 protein level in whole cell lysate (Figure 5C). Conversely, AITC pretreatment 1 h before cyclohexamide treatment for the indicated time markedly increased the protein level and delayed the degradation of NRF2 (Figure 5C), indicating that AITC increases the stability of NRF2 by reducing spontaneous degradation. As described above, Keap1 consistently binds to NRF2 and promotes ubiquitination of NRF2, leading to proteosomal degradation in the normal state. Considering the mechanism of NRF2 regulation and our data (Figure 2 and Figure 5A–C), it is clear that AITC quickly increases the NRF2 protein levels in whole cell lysates by decreasing the degradation of NRF2, and induces nuclear translocation, leading to target gene expression (Figure 5D).

### 3.5. AITC Elevates HO-1 Expression in Mouse Liver Tissues

**Figure 6:**
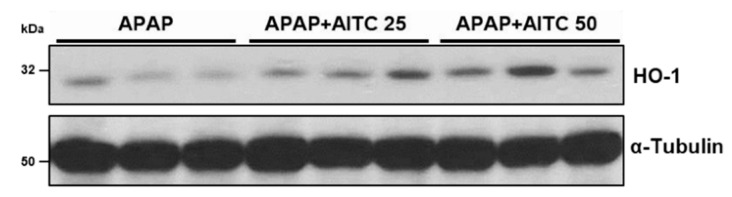
AITC increases HO-1 expression in mouse liver tissues. Mouse liver tissues from the 6 h APAP treatment set were examined by immunoblotting to investigate HO-1 expression.

After the in vitro determination of the effects of NRF2 activation by AITC and its mechanism, we evaluated whether AITC treatment positively regulates the NRF2 target gene in mouse liver, using liver tissues exposed to APAP for 6 h (Figure 6). We observed that both doses of the AITC-treated groups show relatively higher expression of HO-1, as compared with the APAP-only treated group, which is consistent with in vitro results and indicates the potential of AITC as an NRF2 activator in liver.

## 4. Discussion

In our previous work, we reported the protective effect of AITC on intestinal epithelium in DSS-induced colitis model via increasing the tight junction and mucin expression [8]. In this study, we endeavored to examine another beneficial effect of AITC, by investigating the effects of AITC on liver. Studies were targeted using the liver since this is the organ where the orally administered agent first travels to and is metabolized after absorption. When orally administered, AITC is mainly metabolized in the liver via the mercapturic acid pathway, producing GSH-ATIC and NAC-AITC [9]. Therefore, considering the pharmacokinetic property of orally treated AITC, we believed that the liver is one of the most attractive target organs of AITC. Although a previously published work covered the hepatoprotective effect of AITC using CCl_4_ in a rat model [24], their work mainly focused on the Kupffer cell regulation effects of AITC. Little is known regarding the effects of AITC on liver and its mechanism.

APAP overdose is a major cause of acute liver failure in the Western world [31]. At the therapeutic dose, APAP is metabolized by sulfation and glucuronidation, after which it gets excreted in the urine. Meanwhile, a small portion of APAP is metabolized by CYP2E1, producing NAPQI [10]. The amount of NAPQI produced from a therapeutic dose of APAP is usually detoxified by GSH, resulting in no hepatic injury. However, overdose of APAP causes the production of large amounts of NAPQI by CYP2E1, thereby depleting GSH. The excessive NAPQI reacts with cellular proteins in hepatocytes, causing oxidative stress and cell damage [11]. Similarly, murine liver shows acute hepatotoxicity when administered an overdose of APAP. Thus, the APAP-induced hepatotoxicity model is a widely used experimental animal model to evaluate antioxidant reagents or study the role of genes [12,13].

Considering the pathogenesis of the APAP-induced hepatotoxicity model, overdose of APAP primarily induces death of hepatocytes, especially near the central vein region where CYP2E1 is highly expressed. Among the various cell types in the liver, hepatocytes are the major cell type involved in the APAP-induced hepatotoxicity model. Hence, we used the APAP overdose model to investigate the hepatoprotective effects of AITC, focusing on hepatocytes, which constitute a major portion of the liver parenchyma and represent the main cell type metabolizing AITC.

As described above, hepatocytes are the main cell types involved in the APAP overdose model. Nevertheless, hepatocytes primarily damaged by APAP release damage-associated molecular patterns (DAMPs), which in turn result in the activation of innate immune cells and sterile inflammation [29]. Thus, the immune cell involvement in the APAP overdose model is a late time event, and the anti-inflammatory effects of AITC may affect immune cells at a later time. This is one possible explanation for the differing results obtained for the AITC low-dose groups (APAP 6 h or 13 h, Figure 1A,B).

Another possible explanation is the involvement of metabolites produced from AITC in the liver. According to a previous paper covering the pharmacokinetics of AITC and the beneficial effects of its metabolites, GSH-AITC and NAC-AITC are distributed in organs including the liver and kidneys, and are detectable in the liver until at least 8 h (analyzed end time point of the work) after oral administration of AITC [9]. Also, they showed the anti-lipogenic/adipogenic effects of these AITC metabolites using an in vitro system. Although there are few studies reporting the hepatoprotective effects or anti-inflammatory effects of AITC metabolites, the function of AITC metabolites in the liver or kidneys (metabolic organ and excretory organ, respectively) would be an interesting topic for future research.

AITC is known for its various effects, including anti-inflammation, anti-cancer, and anti-oxidation effects [1,2,32]. Notably, the antioxidant effects of AITC have been associated with NRF2 activation [32]. Since APAP-induced cell damage is related to oxidative stress, and the importance of NRF2 in APAP-induced hepatotoxicity has been reported using an NRF2 knockout mouse model [17,18], we confirmed NRF2 activation by AITC, and determined the expressions of its target genes using hepatocyte cell lines. Also, APAP-induced cell damage was observed to diminish via AITC treatment, and the attenuation effect of AITC against APAP was decreased by NRF2 transient knockdown, implying that the protective effects of AITC are dependent on NRF2.

We further investigated the mechanism of NRF2 activation by AITC. According to previous studies, there are several mechanisms that activate NRF2, such as the regulation of NRF2 transcription [33] and Keap1 degradation [34]. In our in vitro experiments, the treatment of HepG2 cells with AITC resulted in rapid increases of NRF2 protein levels in whole cell lysates within 1 h (Figure 5A). However, no remarkable changes were observed at the NRF2 transcription level after AITC treatment for 1 h~12 h (Figure 5B). Conversely, the AITC 1 h pretreated cells showed higher levels of NRF2 and a delayed degradation pattern of NRF2 when incubated with cyclohexamide, as compared to the cyclohexamide-only treated cells devoid of AITC treatment (Figure 5C). Collectively, these results demonstrate that AITC increases the stability and activity of NRF2 by reducing spontaneous degradation. Taken together, we conclude that AITC exerts protective effects on the liver against APAP-induced damage via the NRF2 pathway. We therefore propose the potential of applying AITC as a protective agent targeting the liver through NRF2 activation.
